# Supplementary material for: Flame resistant cotton lines generated by synergistic epistasis in a MAGIC population
Source: PLoS One. 2023 Jan 18;18(1):e0278696. doi: 10.1371/journal.pone.0278696 (PMC9847967; doi:10.1371/journal.pone.0278696)
Supplement: Attachment — Submitted filename: Response to Reviewers-Thyssen-11072022.pdf [file pone.0278696.s014.pdf]

November 7, 2022

Dear Dr. Yuan-Ming Zhang, Reviewers and Editorial Board of PLOS ONE:

I am submitting a twice revised manuscript for your consideration, titled, "**Flame resistant cotton lines generated by synergistic epistasis in a MAGIC population**." [PONE-D-22-19253]

We appreciate your time, consideration, and valuable comments. Below we have responded to each of the new comments and have made additional modifications to the manuscript to reflect your requests and suggestions.

Sincerely,

Gregory Thyssen, Ph.D.

Computational Biologist
Cotton Chemistry and Utilization Unit &
Cotton Fiber Bioscience Unit
USDA-ARS

Reviewer #3:
1. Line 92: remove ref 31.

>>Done.

2. Line 159-162: can you explain more how you did linkage analyses using 4 RILs? It is an haplotype analyses to compare the 4 lines?

>>Added:
"Analysis of the whole genome sequencing was used to identify these common haplotypes by comparing variant call files (VCF), that were generated by samtools and bcftools mpileup software, with bedtools intersect software [39]."

3. Line 249-254: In table S4, could you add those loci within a few Mb from PLINK or mrMLM?

>>We have added a new Table S5 that includes **all** the RNAseq expression data for **every** annotated gene with any detectible expression (>0 RPKM) (N=41,746) in the 8 RILs at developmental stages of 8-DPA and 16-DPA.

4. Line 256-264: could you describe more about any of those 13 genes are potential candidates or consistent with any other published research? I understand that the flammable traits may not have may published results. Candidate for any traits highly correlated with it? You mentioned some in the discussion but you may want to explain more in the main text.

>>The supplemental Tables S5-S10 contain the gene descriptions and identification of the Arabidopsis orthologs for the candidate genes, differentially expressed genes and all expressed genes. The first

version of this manuscript did include more speculation in the discussion and main text about flavonoid biosynthesis genes among the candidates, (a pathway we previously found to be relevant in brown cotton with natural flame retardance) but the previous reviewers at PLOS One suggested that we reduce that speculation.

5. Line 290: using highest or lowest HRC values instead of worst.

>>Done.

6. Line 327: remove “Found among”, and “were lines that”.

>>Done.

7. Line 335: remove “already”.

>>Done.
