# Supplementary material for: Flame resistant cotton lines generated by synergistic epistasis in a MAGIC population
Source: PLoS One. 2023 Jan 18;18(1):e0278696. doi: 10.1371/journal.pone.0278696 (PMC9847967; doi:10.1371/journal.pone.0278696)
Supplement: Attachment — Submitted filename: Response to Reviewers-Thyssen-08102022.docx [file pone.0278696.s013.docx]

August 10, 2022

Dear Dr. Yuan-Ming Zhang, Reviewers and Editorial Board of PLOS ONE:

I am submitting a revised manuscript for your consideration, titled, “**Flame resistant cotton lines generated by synergistic epistasis in a MAGIC population**.” [PONE-D-22-19253]

We appreciate your time, consideration, and valuable comments. Below we have responded to each of the comments and have made modifications to the manuscript to reflect your requests and suggestions.

Sincerely,

Gregory Thyssen, Ph.D.

USDA-ARS

***Comments to the Author:***

*2. Has the statistical analysis been performed appropriately and rigorously?*

*Reviewer #1: No*

*Reviewer #2: No*

**RESPONSE:**

All p-values shown in the Tables and Supplemental Tables were computed using the default statistical methods implemented by the highly cited bioinformatic software described in the Methods. No attempt was made to artificially reduce thresholds for significance or to employ other-than-default statistical analysis. Rather than presenting just one of the genomic analyses, we present three (PLINK, mrMLM, simple linkage), which do not completely agree with each other. We agree that the genomics part of this report is preliminary, so we believe that presenting all of these data will be the most beneficial to the other researchers and breeders that will hopefully help continue this work, so that naturally flame-resistant cotton can be further characterized and used in breeding programs.

***Reviewer #1****:*

*In this manuscript entitled “Flame resistant cotton lines generated by synergistic epistasis in a MAGIC population” presented the identification of the lower heat release capacities textiles made from cotton fiber produced from MAGIC RILs. The flame-resistant trait of cotton germplasm has the potential to greatly reduce the costs of flame retardant additives for cotton textiles. To determine the genetic mechanism of the flame-resistant cotton by transgressive segregation, the authors used the GWAS and RNAseq methods, identified 26 loci and 13 genes were linked to the influence the HRC values in the 257 RILs. The authors showed an interesting topic, however, there are some concerns and suggestions for authors to address:*

*1. How about the HRC values of the eleven parents of MAGIC? The authors should show the experiments and results in the MS.*

**RESPONSE:**

The HRC values of the eleven parents are represented by the vertical black lines in the histogram in Figure 1a. Now, we have added mention of the parents in the Methods and a new Supplemental Table 1 with the numerical values.

*2. Whether there is a difference in the flavonoid content between the flammable lines and FR lines? to confirm the previous suggestion that a colorless flavonoid or other natural colorless compound was responsible for the FR. And to support the GWAS and RNAseq data.*

**REPSONSE:**

Quantification of flavonoid compounds and other metabolites in the flame resistant and flammable lines is ongoing. Unfortunately, due to the COVID pandemic, our collaborator that was working on this has not yet been able to complete the study. We may need to grow another season for fresh material and/or replication of the experiment. Now, we have modified the manuscript to emphasize that the flavonoid mechanism remains at this point a hypothesis for future work and not a claim of a finding. As mentioned above, publication of this work will hopefully encourage other groups to test the material and develop additional samples, hypotheses, and findings.

*3. The GWAS and RNAseq results are too preliminary, it does not make a whole story.*

**RESPONSE:**

We agree that the genomics part of this report is preliminary. Rather than work in isolation to discover the complete mechanism, we choose to share the candidate genes and loci that we could find already by various approaches. Since the mechanism is likely the synergistic interaction of numerous small-effect loci, much further work will be required to present the whole story. However, these FR lines already have value to breeders since phenotypic selection by MCC is feasible at scale. Now, we acknowledge and further emphasize these points in the Conclusion. Again, exposure of our findings to other researchers and breeders is the fastest way for the whole story to be discovered.

*4. To further focus on the 41 segregated for non-synonymous variants in the RMUP-MAGIC population among the 602 unique genes, and the 13 highly differentially expression genes within the locations, to determine the genetic mechanism of the flame-resistant cotton.*

**RESPONSE:**

We do intend to further focus on our preliminary candidate genes to identify the whole story of the mechanism, and to develop genetic markers for marker assisted breeding. However, this requires another growing season, so that more stages of the developing cotton fiber tissues can be collected for RNA extraction and quantification. We are also currently developing additional informative populations to isolate and study subsets of the preliminary candidate genes. Publication of the present study will hopefully lead to study of these lines and our currently identified FR RILs at additional locations, both by our collaborators and other independent colleagues in cotton research and breeding.

***Reviewer #2:***

*The natural flame resistant is an important trait in cotton. Linkage, epistatic, GWAS and transcriptomes analysis were conducted to determine the genetic architecture in MAGIC population of cotton. Of the textiles fabricated from the five offspring of MAGIC, four exhibited the novel characteristic of inherent flame resistance. It is expected to provide guidance for breeding. This is an interesting study. But I have some concerns in statistical analysis.*

*1. The greatest advantages of QTL mapping using MAGIC populations come from multiple founder alleles. Many researchers think that multi-allele model should be considered in the statistical analysis of MAGIC population. Multi-allele model can estimate the effect of every allele and it is beneficial to crop breeding. In this study the GWAS method was used to analyze the datasets of 11-parent MAGIC population, but all GWAS methods consider only two alleles. Please explain why the multi-allele model did not use.*

**RESPONSE:**

*Gossypium hirsutum* cultivars have limited genetic diversity and a narrow genetic base, especially when compared to other crops like *Zea mays*. In this population, within the 473,000 high quality SNPs, there are no multi-allelic SNPs. Now, the manuscript has been modified to highlight this fact. Further analysis to explore the utility of a multi-allelic analysis could be achieved by first identifying haplotype blocks or regions of identity-by-descent that are large enough to include enough polymorphic SNPs to define multiple alleles of each haplotype. So far, we have not found such, but we agree with the suggestion and will continue to explore multi-allele models in future work.

*2. When the GWAS method was used to analyze the dataset of MAGIC population, please consider whether the population structure should be considered.
These reasons may make your results difficult to understand. For example, the loci identified in linkage analysis are apart from the loci identified by PLINK and/or mrMLM.*

**RESPONSE:**

This structure of this MAGIC population has been characterized several times. First with 1,600 SSR markers, then with 6,000 SNP markers, and again with the 473,000 SNPs identified by whole genome sequencing used in the present study. [Refs 31, 32, 28 in manuscript]. These all indicated that the five cycles of bulked-pollen random mating, followed by six generations of self-pollination, was successful at creating a population with no significant population structure.

We hypothesize that the discrepancies between PLINK and mrMLM are due to the inherent difficulty in identifying the mechanism of a trait that is controlled by numerous small effect loci and synergistic epistatic interactions. Since none of the parent lines possess the flame resistance phenotype, elucidation of the mechanism is not straightforward. We believe that presenting the readers with the multiple genetic analyses, though some loci differ between the approaches, is more useful for the advancement of the research by the larger cotton research community than to stretch to reconcile them, at this point.
